# Differential Effects of In Vitro Simulated Digestion on Antioxidant Activity and Bioaccessibility of Phenolic Compounds in Purple Rice Bran Extracts

**DOI:** 10.3390/molecules29132994

**Published:** 2024-06-24

**Authors:** Pitchaporn Wanyo, Tossaporn Chamsai, Nitchara Toontom, Le Ke Nghiep, Kukiat Tudpor

**Affiliations:** 1 Department of Food Technology, Faculty of Agricultural Technology, Kalasin University, Kalasin 46230, Thailand; pitchaporn.wa@ksu.ac.th; 2 Department of Mechanical Engineering, Faculty of Agriculture and Technology, Rajamangala University of Technology Isan, Surin Campus, Surin 32000, Thailand; tossaporn_c@kkumail.com; 3 Public Health and Environmental Policy in Southeast Asia Research Cluster (PHEP-SEA), Mahasarakham University, Maha Sarakham 44150, Thailand; kukiat.t@msu.ac.th; 4 Faculty of Public Health, Mahasarakham University, Maha Sarakham 44150, Thailand; 5 Vinh Long Department of Health, Vĩnh Long 85000, Vietnam; lekenghiep@gmail.com

**Keywords:** pigmented rice brans, in vitro simulated digestion, phenolic compounds, antioxidants, bioaccessibility

## Abstract

Pigmented rice varieties are abundant in phenolic compounds. Antioxidant activity and bioaccessibility of phenolic compounds are modified in the gastrointestinal tract. After in vitro simulated digestion, changes in antioxidant activity and bioaccessibility of phenolic compounds (phenolic acids, flavonoids, and anthocyanins) in purple rice brans (Hom Nil and Riceberry) were compared with undigested crude extracts. The digestion method was conducted following the INFOGEST protocol. Antioxidant activity was determined using the ferric-reducing antioxidant power (FRAP) and 2,2-diphenyl-1-picrylhydrazyl (DPPH) radical scavenging activity assays. The bioaccessibility index (BI) was calculated from the ratio of digested to undigested soluble phenolic content. Overall results showed that the in vitro simulated digested rice brans had lower antioxidant activity and lower total phenolic, flavonoid, and anthocyanin contents. However, the concentration of sinapic acid was stable, while other phenolic acids (gallic, protocatechuic, vanillic, *ρ*-coumaric, and ferulic acids) degraded after the oral, gastric, and intestinal phases. The BI of sinapic, gallic, vanillic, and ferulic acids remained stable, and the BI of quercetin was resistant to digestion. Conversely, anthocyanins degraded during the intestinal phase. In conclusion, selective phenolic compounds are lost along the gastrointestinal tract, suggesting that controlled food delivery is of further interest.

## 1. Introduction

It has been well established that oxidative stress plays a pivotal role in the etiology of chronic diseases, including obesity, diabetes, hypertension, and cardiovascular diseases [1]. Bioactive compounds with antioxidative properties in new food products and nutraceuticals can provide health benefits against these diseases [2]. In this regard, purple rice (*Oryza sativa* L.) belongs to the pigmented rice group and is a rare variety of rice that accumulates anthocyanins in its seed coat [3]. Purple rice has also been a traditional food and medicinal plant for a long time [4]. Among the varieties, Hom Nil and Rice-berry, two strains developed in Thailand, are noted for their distinctive purple hue attributed to high levels of anthocyanins. Hom Nil, non-glutinous pigmented rice, has been reported as a good source of antioxidants containing phenolic compounds [5]. Riceberry is crossbred between Hom Nil and Khao Dawk Mali 105 varieties [6]. These rice varieties have a deep purple color because they contain large amounts of anthocyanin pigments, especially in the pericarp, a part of rice bran [7], and are becoming popular in the global market because of their potential health benefits [8]. In Thailand, rice bran is widely used as animal feed, and its price is low [6,8]. Purple rice bran contains many bioactive compounds, including anthocyanins and other phenolic acids and flavonoids, which are compounds known for their potent antioxidant properties. Nevertheless, the bioactivity of these compounds is subject to environmental changes, such as light intensity, temperature, oxygen, pH, and, when ingested, human gastrointestinal conditions [9,10,11]. Therefore, total bioactive compounds and other natural antioxidants in food do not always reflect the total amount available to be absorbed and metabolized by the human body. Previous research has shown that purple rice anthocyanins and phenolic compounds have promising digestion properties and synergistic effects on biological activity after simulated gastrointestinal digestion in vitro [9,10,11].

Food components are constantly exposed to mechanical and chemical processes in the gastrointestinal digestion—oral, gastric, and intestinal phases. The oral phase of digestion begins with a short period of physical breakdown of solid foods by chewing, forming a paste-like bolus before swallowing, and has no discernible effect on digestion [12]. In the gastric phase, a person perceives foods through multisensory perception—sight, taste, smell, associations, memory, emotions, and hedonics of foods (a.k.a. the cephalic phase)—activating the dorsal motor nucleus of the vagus nerve in the medulla oblongata [13]. These sensory inputs result in gastric secretion in preparation for digestion. When the bolus enters the stomach, it activates stretch receptors in the stomach wall and chemoreceptors in the mucosa, resulting in more gastric secretion [13]. The intestinal phase possesses complex enzymatic digestion of macronutrients with proteases, amylase, and lipases [13]. It is noteworthy that the structures and functions of some phytochemicals are modified in the intestine. Friedman and colleagues showed that caffeic, chlorogenic, and gallic acids were irreversibly stable to high pH. Contrarily, chlorogenic acid was resistant to acid pH and temperature. In addition, catechin, epigallocatechin, trans-cinnamic acid, ferulic acid, and rutin were stable in a high pH milieu [14].

However, the antioxidant activity and bioaccessibility in gastrointestinal environments have not been determined. Therefore, this study aimed to investigate the effects of the in vitro simulated gastrointestinal digestion on antioxidant activity and bioaccessibility of phenolic acids, flavonoids, and anthocyanins in two purple rice brans (Hom Nil and Riceberry). Our experiments aim to provide valuable information that could help optimize plant-based bioactives and promote the utilization of natural resources for improving human health by analyzing the stability and bioaccessibility of phenolic compounds during digestion.

## 2. Results and Discussion

### 2.1. Antioxidant Activity

**Figure 1:**
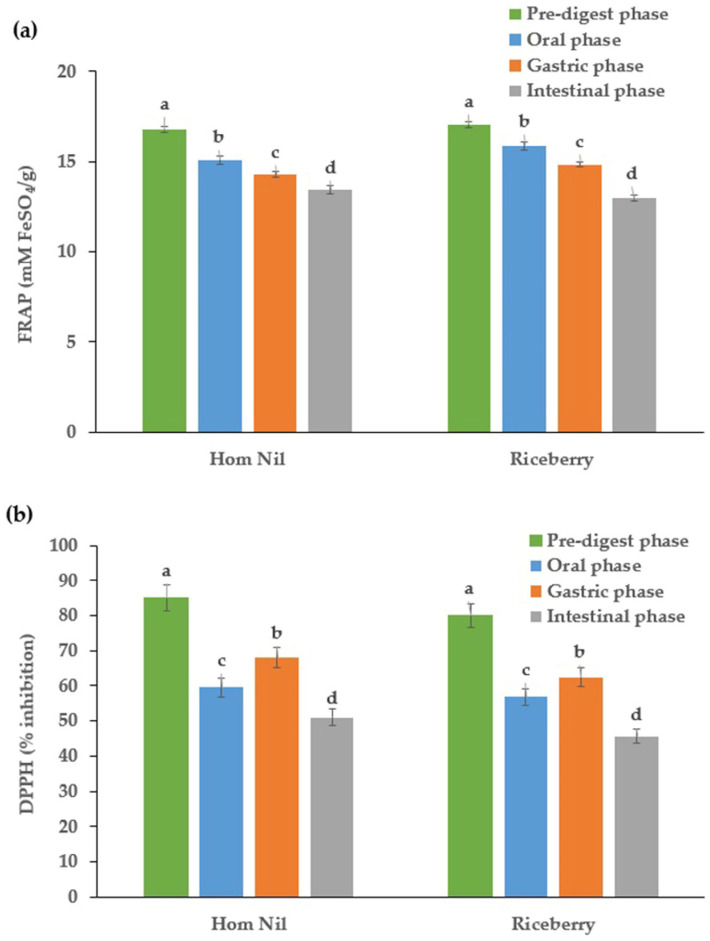
Effect of simulated in vitro digestion on (**a**) ferric-reducing antioxidant power and (**b**) DPPH radical scavenging activity of purple rice bran extracts. Bars are expressed as means ± standard deviation (*n* = 3). Different letters in the same rice bran variety of the in vitro digestion stages were significantly different at *p* < 0.05.

The antioxidant activity of purple rice bran extracts was determined using DPPH and FRAP assays. These distinct methods were chosen to correctly evaluate the antioxidant activity of the extracts, as they have different action mechanisms, and the responses of the individual phenolic compounds differ between them [15]. Changes in antioxidant activity during the simulated in vitro gastrointestinal digestion of purple rice bran extracts are presented in Figure 1. FRAP values of both Hom Nil and Riceberry rice bran extracts revealed a gradual decrease during in vitro digestion. As shown in Figure 1a, the FRAP values decreased from the initial crude extracts (16.78 and 17.07 mM FeSO_4_/g in Hom Nil and Riceberry, respectively) to the intestinal digestion phase (13.43 and 12.97 mM FeSO_4_/g in Hom Nil and Riceberry, respectively), indicating reductions of 19.98% and 24.00%, respectively. From Figure 1b, the DPPH value of Hom Nil rice bran extract was highest in the initial crude extracts (85.06%) and decreased through the digestion stages: stomach (59.54%) and small intestine (51.04). Similarly, the Riceberry extract started with a DPPH inhibition of 80.09% and ended with 45.65% after intestinal digestion, showing a reduction of 43.00%.

After gastric digestion, the antioxidant activity of the extracts showed a significant decrease, which is attributed to the degradation of phenolic compounds in the acidic environment of the stomach. This trend is consistent with previous studies indicating that phenolic compounds are susceptible to pH changes during digestion [16,17]. A notable decrease was observed upon digestion, possibly due to the degradation or transformation of phenolic compounds during the simulated gastrointestinal digestion. Similar findings were also observed by Peanparkdee et al. [11], who reported that the activities from Thai rice bran extracts measured by DPPH and FRAP assays decreased after simulated digestion. José Jara-Palacios and colleagues evaluated the effects of in vitro gastrointestinal digestion on the antioxidant activity of different white winemaking by-product extracts [18]. It was found that the DPPH value of the winemaking by-product decreased from undigested samples to gastric digests, although a slight increase was found from the gastric phase to the intestinal phase. However, the final values of antioxidant activity were much lower than those for undigested samples. Elejalde and co-workers also reported that grape seed extracts’ DPPH radical scavenging activity decreased from undigested samples to the intestinal phase, with final antioxidant capacity values much lower than those for undigested samples [19].

Notably, the radical scavenging activity evaluated by in vitro antioxidant assays alone does not emphasize the potential systemic benefits of phenolic compounds to health. The levels of polyphenols in the gastrointestinal tract during the digestion of food serve as the direct antioxidant effect, suggesting that the bioavailability of these compounds might be reduced after ingestion, potentially affecting their health benefits when consumed [20]. In different stages of digestion, the number of polyphenol antioxidants released from complex food systems varies. For example, during the oral stage, the conversion of catechin and epicatechin occurs due to the degradation reaction of microbes and enzymes [21]. Due to the short contact time with the enzyme, the polyphenols release a quantity significantly lower than in the subsequent stages of the stomach and intestine under the stimulus of acidic gastric juice [22]. Moreover, the plant matrix affects the retention rate of specific phytochemicals [23]. Therefore, the food matrix and the composition of phytochemicals may also be responsible for the differences in the antioxidant activity of different foods after in vitro digestion.

### 2.2. Phenolic Content

**Figure 2:**
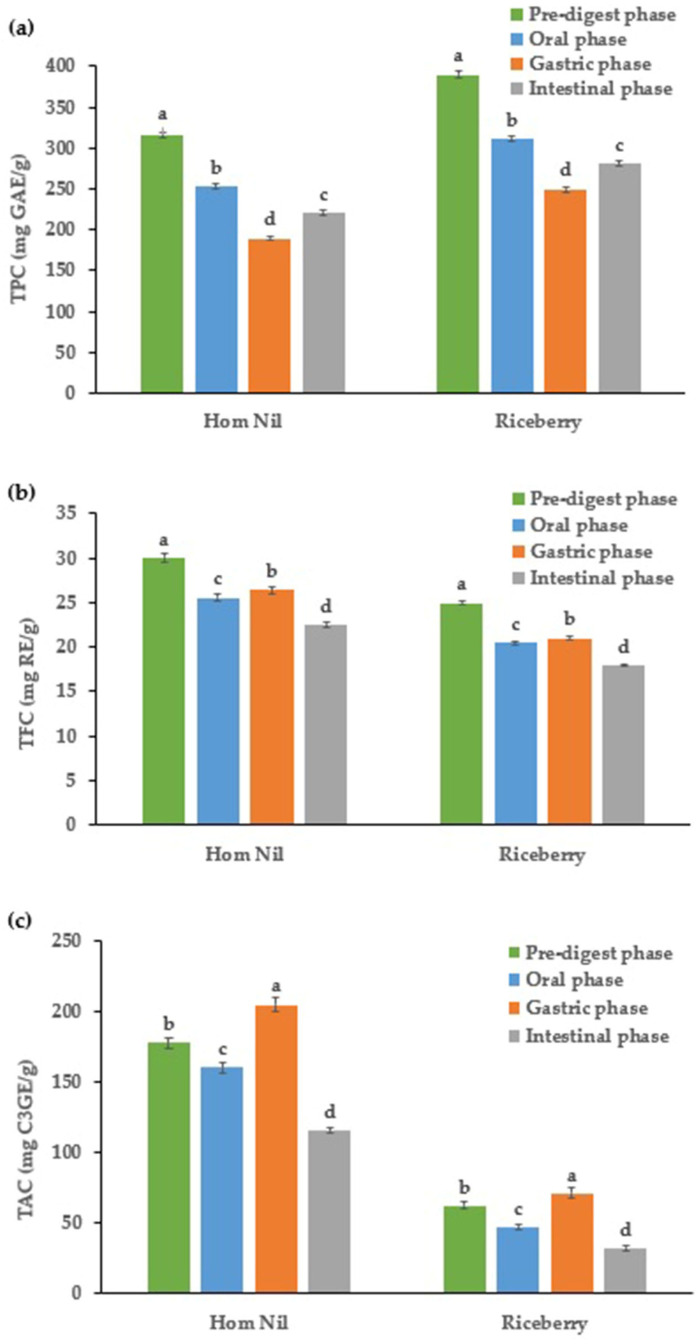
Effect of simulated in vitro digestion on (**a**) TPC, (**b**) TFC, and (**c**) TAC of purple rice bran extracts. Bars are expressed as means ± standard deviation (*n* = 3). Different letters in the same rice bran variety of the in vitro digestion stages were significantly different at *p* < 0.05.

The results of the phytochemical simulation for the in vitro digestion of purple rice bran extracts, total phenolic content (TPC), total flavonoid content (TFC), and total anthocyanin content (TAC), are shown in Figure 2. Figure 2a shows that Hom Nil and Riceberry declined the TPC throughout digestion. Hom Nil’s TPC decreased from 315.97 mg GAE/g dry weight in the crude extract to 221.18 mg GAE/g dry weight after the intestinal phase. Similarly, Riceberry’s TPC dropped from 390.02 mg GAE/g dry weight to 280.81 mg GAE/g dry weight, representing a decrease of 28.00%. These are per previous reports on Thai rice bran extracts [11], purple rice [9], grape seed [18,19], and grape pomace extracts [18]. From Figure 2b, the TFC also reduced upon digestion, with Hom Nil’s TFC going from 30.04 mg RE/g dry weight in the crude extract to 22.53 mg RE/g dry weight in the intestinal phase, a reduction of about 25.00%. Riceberry exhibited a reduction from 24.97 mg RE/g dry weight to 17.98 mg RE/g dry weight, which is a decrease of 28.00%. Barak and colleagues reported slightly reduced total flavonoid content in European cranberry (*Viburnum opulus* L.) fruit extracts [24].

On the other hand, Quan and colleagues observed that after the digestive process, the content of total flavonoids increased by 4.62 and 3.30-fold for the pulp and seed of raspberry, respectively [25]. Bouayed and colleagues suggested that the decrease in TPC and TFC in digested plant samples could be attributed to the decomposition of polyphenols, such as anthocyanin, which are sensitive to an alkaline pH environment in the intestinal digestion phase [26]. As shown in Figure 2c, the TAC of Hom Nil decreased significantly, starting at 178.25 mg C3GE/g dry weight and ending at 115.86 mg C3GE/g dry weight, representing a reduction of approximately 35.00%. Riceberry’s TAC started higher at 62.48 mg C3GE/g dry weight and finished at 32.44 mg C3GE/g dry weight, showing a decrease of 48.08%. This reduction aligns with previous studies on Thai rice bran extracts, purple rice, grape seed, and grape pomace extracts [9,11]. The consistent reduction across these phytochemical groups suggests that digestive enzymes and pH changes contribute to the breakdown or modification of these compounds, impacting their bioaccessibility. However, the health benefits of these compounds may also be derived from the metabolites formed post-digestion.

The results demonstrate a clear trend of decreasing TPC, TFC, and TAC after the digestion process for both varieties of rice brans, suggesting that the bioactive compounds degrading phytochemicals were altered, leading to a reduced ability to quantify them. The digestive enzymes and the stomach’s acidic environment, followed by the conditions in the intestine, might contribute to the breakdown or modification of these phytochemicals, impacting their extraction and measurement [21,27]. The consistent reduction across all three phytochemical groups indicates a general trend of reduced bioaccessibility of these compounds after digestion. However, the health benefits of these compounds may not solely be from the compounds themselves but also from their metabolites formed after digestion. Therefore, it is crucial to understand the metabolism and bioavailability of both the parent compounds and their metabolites.

### 2.3. Qualitative and Quantitative Composition of Phenolic Compounds

The individual phenolic compounds in the purple rice bran extracts, Hom Nil and Riceberry, throughout different stages of simulated in vitro digestion, are shown in Table 1. Concentrations of phenolic acids, flavonoids, and anthocyanins were measured in milligrams per gram (mg/g) at the crude extract (pre-digest), oral, gastric, and intestinal phases. After simulated gastrointestinal digestion, the amount of each phenolic acid and flavonoid in both rice bran extracts was lower than in crude extracts. For the Hom Nil variety, total phenolic acids decreased progressively from the crude extract (3.97 mg/g) to the intestinal phase (2.88 mg/g). A similar trend was observed with total flavonoids, reducing from 4.31 mg/g in the crude extract to 3.15 mg/g after intestinal digestion. For the Riceberry variety, a reduction in total phenolic acids from the crude extract (4.14 mg/g) to the intestinal phase (3.04 mg/g) was observed. There was a decrease in total flavonoids from the initial crude extract (3.97 mg/g) to the intestinal digestion stage (2.86 mg/g). Regarding anthocyanins, the total amount of anthocyanins in the Hom Nil rice bran extract displayed an initial decrease during the oral and gastric phases while it increased during the small intestinal phase before dropping significantly in the intestinal phase (from 157.72 mg/g in SSF to 71.58 mg/g in SIF). For Riceberry, the pattern was similar to that of Hom Nil, with an increase in the small intestinal phase and a significant reduction after intestinal digestion (from 60.05 mg/g in SSF to 29.23 mg/g in SIF).

The results highlight the dynamic changes that phenolic compounds undergo during digestion. The overall trend for rice bran varieties is a reduction in phenolic compounds as digestion progresses. Digestive enzymes may alter the structure of phenolic compounds, reducing their detectability or extraction efficiency. Changes in pH throughout the digestion stages can impact the stability of phenolic compounds, particularly anthocyanins, which are known to be sensitive to pH variations. The pH levels in the intestine and the effect of bile salts lead to modifications in chemical structures. This, in turn, produces new compounds that possess bioavailability and biological activities [7,22]. Polyphenols undergo metabolism by the microbiome, producing compounds that may have bioactive properties. It is important to note that phenolic compounds with antioxidant capacity, which remain stable in acidic pH during the gastric phase, may not necessarily retain this capacity in the neutral or alkaline pH of the intestinal phase, indicating that pH influences polyphenol stability and the possibility of degradation or conversion of these compounds [28]. Anthocyanin stability involves the changes in their physicochemical properties during the digestive processes in the gastrointestinal tract [29]. The liberation of anthocyanins from plant cell vacuoles is crucial before their absorption as bioactive compounds.

Various factors influence the stability and bioavailability of anthocyanins from purple rice bran extracts during digestion. Anthocyanins, sensitive to pH changes, can degrade significantly in the digestive tract, particularly in the acidic gastric phase. This degradation can affect their bioavailability, which refers to the portion of digested anthocyanins that enter the systemic circulation and become accessible to various tissues and organs, thereby exerting their biological effects. The digestion process and the food matrix can influence the release and stability of anthocyanins. For instance, studies have shown that anthocyanins are more stable in acidic environments, such as the stomach, which could explain their significant reduction in content during the intestinal phase of digestion [9,29]. Therefore, understanding the factors that affect anthocyanin stability and bioavailability is crucial for optimizing their health benefits [30].

### 2.4. Bioaccessibility of Phenolic Compounds

The bioaccessibility index of selected phenolic compounds in purple rice bran extracts (Hom Nil and Riceberry) through simulated in vitro digestion phases is shown in Table 2. Phenolic acids in Hom Nil rice bran extract, gallic, protocatechuic, vanillic, *ρ*-coumaric, ferulic, and sinapic acids showed high bio-accessibility during the oral phase, although the BI for ferulic acid was 50%, which is relatively lower compared to others. Protocatechuic and *ρ*-coumaric acids exhibit significant declines from the oral to the intestinal phase. Riceberry shows a trend similar to Hom Nil, with high bioaccessibility in the oral phase and a decrease through the gastric and intestinal phases. Gallic and protocatechuic acids demonstrate notable reductions in the intestinal phase.

Interestingly, ferulic and sinapic acids were not significantly altered throughout the phases of digestion. Notably, ferulic acid in black carrots was stabilized throughout the digestion [31]. A previous study by Friedman and Jürgens showed that ferulic acid was stable at pH 7 to 11 due to its molecular structure with a single OH group [14]. Along with sinapic acid, ferulic acid stabilized anthocyanins in strawberry juice, emphasizing their stable properties [32].

Flavonoids in both Hom Nil and Riceberry, including rutin, myricetin, and quercetin, also start with relatively high bioaccessibility in the oral phase, with a general trend of decreasing bioaccessibility through the gastric and intestinal phases. The results were supported by the recent finding by Elejalde and colleagues that the diminished bioaccessibility from the oral to the intestinal phase observed in purple rice bran phenolics is similar to grape phenolics [19]. In both cases, the initial high bioaccessibility of phenolics decreases through subsequent digestive phases. However, the decrease in bioaccessibility was not as pronounced as that observed for quercetin, which commonly undergoes autooxidation and degradation under alkaline pH [33]. Its stability in the intestinal phase was probably due to its synergistic complex matrix formation with other phenolic compounds [34]. Anthocyanins showed a significant increase in the gastric phase for cyanidin 3-glucoside, peonidin 3-glucoside, and malvidin 3-glucoside, but a decrease was evident in the intestinal phase.

The results indicate that phenolic compounds in purple rice brans are initially highly bioaccessible because they are released from the food matrix during chewing. However, as digestion progresses, their bioaccessibility tends to decrease due to enzymatic degradation, changes in pH, and interactions with other food components or digestive enzymes, which can reduce their solubility or stability [22]. Pancreatin, which contains lipases, amylases, and proteases, may contribute to the bioaccessibility of phenolic compounds [35]. The increase in bioaccessibility of some anthocyanins during the gastric phase might be attributed to the stomach’s acidic environment, which can stabilize anthocyanins and enhance their solubility. However, the subsequent decrease in the intestinal phase could result from multiple factors, including enzymatic breakdown, complexation, and increased pH. Pineda-Vadillo et al. concluded that anthocyanins remain stable and soluble during the oral and gastric stages of digestion, whereas many of them are extensively modified or insolubilized during the intestinal stage of digestion [36]. The variability of phenolic compounds suggests that each compound may have its unique stability profile and interaction with digestive components, affecting its bioaccessibility. These results indicate that although purple rice bran is rich in bioactive compounds, the benefits of consuming these compounds could be influenced by how easily they are absorbed and how stable they remain during digestion. It is important to note that the bioaccessibility index does not account for the absorption and metabolism of these compounds, which are critical to understanding their actual bioavailability and potential health benefits. The bioaccessibility data can guide further studies on enhancing the stability and absorption of phenolic compounds in the gastrointestinal tract.

## 3. Materials and Methods

### 3.1. Rice Brans and Reagents

Purple paddy rice of Hom Nil and Riceberry cultivars were collected from fields located in Thailand during the 2021–2022 growing season. The bran part was separated from the paddy by a dehusking machine (NW 1000 TURBO, TEP Innovation, Nathawee, Thailand) and stored at −20 °C in aluminum/polyethylene bags until use. α-Amylase from porcine pancreas, pepsin from porcine gastric mucosa, pancreatin and lipase from porcine pancreas, and porcine bile salt were purchased from Sigma-Aldrich (St. Louis, MO, USA). Standard solutions of phenolic compounds [phenolic acid (gallic, protocatechuic, vanillic, *ρ*-coumaric, ferulic, and sinapic acids), flavonoid (rutin, myricetin, and quercetin), and anthocyanin (cyanidin 3-glucoside, peonidin 3-glucoside, and malvidin 3-glucoside)] were purchased from Sigma-Aldrich. Other reagents and chemicals used in the experiments were of analytical grade.

### 3.2. Extraction of Antioxidants from Rice Bran

Five grams of rice bran powders were dissolved in 100 mL of 65% (*v/v*) aqueous ethanol. The mixtures were then sonicated (40 kHz) in an ultrasonic cleaner (POWERSONIC 405; Hwashin Technology Company, Gyeongsangbuk-do, Republic of Korea) at 60 °C for 60 min, as described by Peanparkdee and colleagues with some modifications [37]. After extraction, all solutions were centrifuged at 2500 rpm for 20 min. The supernatant was freeze-dried, resulting in the powder extract. The crude rice bran extracts were stored in a brown bottle at −20 °C until further analysis.

### 3.3. In Vitro Simulated Digestions

The in vitro digestion method followed the INFOGEST protocol described by Brodkorb et al. and Peanparkdee et al. with some modifications [38,39]. It consisted of a three-step procedure mimicking the oral, gastric, and intestinal digestive processes. For static in vitro digestion methods, the experimental conditions are constant during each phase. Simulated saliva fluid (SSF) was prepared by dissolving 0.24 g Na_2_HPO_4_, 0.02 g KH_2_PO_4_, and 0.80 g NaCl in 100 mL distilled water, then adjusting the pH to 6.8. α-amylase was added to the mixture to obtain an enzyme activity of 200 U. Simulated gastric fluid (SGF), 0.32 g pepsin (from porcine gastric mucosa: pepsin A) was mixed with 0.6 mL HCl, and 100 mL of 0.03 M NaCl, and the pH was then adjusted to 1.2 with 1 M HCl. Simulated intestinal fluid (SIF) was obtained by dissolving 0.14 g pancreatin and 0.86 g porcine bile extract in 100 mL of 0.1 M NaHCO_3_, then adjusting the pH to 7.4 with 0.1 M NaHCO_3_. The extracts were digested in three steps—oral phase: rice bran extracts (0.1 g) were mixed with 10 mL of SSF (with α-amylase), and the mixtures were incubated for 2 min in a 37 °C shaking water bath at 120 rpm; gastric phase: the samples from the oral phase were adjusted to pH 2.0 with 5 M HCl, and 10 mL of SGF (with pepsin) was added. The mixtures were continuously shaken for 2 h in a 37 °C shaking water bath; and intestine phase: the pH of digested samples obtained from the gastric phase was adjusted to 7.0 with 1 M NaOH, and 9 mL of SIF (containing pancreatin and bile salt) was added. Then 1.5 mL of NaCl solution and 1.5 mL of KCl solution were added, and the mixtures were kept in a 37 °C shaking water bath for 2 h. The enzymatic reactions were ended with iced water. Aliquots of the digested products were collected at the end of each digestion process and centrifuged at 2500 rpm for 10 min. The supernatants were then separated and stored at −20 °C until further analysis. The simulated digestions were performed in triplicate.

### 3.4. Determination of Antioxidant Activity

The antioxidant activity at each digestion stage was determined using the ferric-reducing antioxidant power (FRAP) and 2,2-diphenyl-1-picrylhydrazyl (DPPH) radical scavenging activity assays, as previously described by Wanyo et al. [40]. Absorbances were measured on a spectrophotometer (Libra S22, Biochrom, Cambridge, UK). For the FRAP assay, the FRAP reagent (1.8 mL), Milli-Q water (180 μL), and the extract (60 μL) were mixed in test tubes. The mixture was incubated at 37 °C for 4 min. The absorbance was read at 593 nm, and the FRAP value was expressed as mM FeSO_4_ per g of dry weight (mM FeSO_4_/g). For the DPPH analysis, the extract (0.1 mL) was mixed with 0.1 mM DPPH in ethanol (2.9 mL) and vortexed (1 min). The mixture was allowed to settle undisturbed (30 min), after which its absorbance was read at 517 nm. The percent inhibition was calculated using the following formula:

$$DPPH\;inhibition\;\left(\%\right)=(\frac{A_{0}-A_{e}\;}{A_{0}})\times 100$$

where *A*_0_ is the absorbance of the DPPH solution, and *A_e_* is the absorbance of DPPH with extract.

### 3.5. Determination of Phytochemical Content

The phytochemical content at each digestion stage was determined in terms of total phenolic content (TPC), total flavonoid content (TFC), and total anthocyanin content (TAC). Absorbances were measured on a spectrophotometer (Libra S22, Biochrom). The TPC and TFC were determined according to the method described by Wanyo et al. [40]. The absorbance was read at 725 nm for TPC, and results were expressed as mg gallic acid equivalents per g of dry weight (mg GAE/g). For TFC, the absorbance was read at 510 nm, and results were expressed as mg rutin equivalents per g of dry weight (mg RE/g). The TAC was measured using a pH differential assay described by Buran et al. [41]. Absorbance at 520 and 700 nm was measured on a UV/Vis spectrophotometer after 15 min of incubation at room temperature. Absorbance (A) was calculated using (A_520_ − A_700_)_pH1.0_ − (A_520_ − A_700_)_pH4.5_. Total anthocyanin content was calculated using (A × 449.0 × 80 × 1000)/(29,740 × 1), and results were expressed as milligram cyanidin 3-glucoside equivalent per gram (mg C3GE/g).

### 3.6. Identification and Quantification of Phenolic Compounds

A high-performance liquid Chromatography (HPLC) system with LC-20AC pumps and SPD-M20A diode array detector (Shimadzu, Japan), and Inetsil ODS-3, C18 (4.6 mm × 250 mm, 5 μm) was used to characterize phenolic compounds. The individual phenolic acid and flavonoids mobile phase consisted of purified water with acetic acid (pH 2.74) (solvent A) and acetonitrile (solvent B) at a flow rate of 0.8 mL/min. The gradient elution conditions used were described previously by Wanyo et al. [40]. The operational settings were a column temperature of 38 °C, an injection volume of 20 µL, and UV-diode array detection at 280 nm for hydroxybenzoic acids and 320 nm for hydroxycinnamic acids. Phenolic compounds in the samples were identified by comparing their retention times and UV spectra to those of standard compounds, using an external standard method for detection. The results were expressed as mg per g dry weight (mg/g).

For the anthocyanins, the mobile phases used were 0.1% hydrochloric acid in methanol (15:85 *v/v*) (phase A) and 8% formic acid (phase B) at a flow rate of 1 mL/min. The gradient elution conditions used were described previously by Duangkhamchan and Siriamornpun [42]. The operating conditions were as follows: column temperature of 30 °C, injection volume of 20 μL, and a detection wavelength of 520 nm. Solutions were injected after being filtered through a 0.45 μm nylon membrane filter. Anthocyanins in the samples were identified by comparing their relative retention times and UV spectra with those of standards and were detected using an external standard method. The results for the anthocyanins were expressed as mg per g dry weight (mg/g).

### 3.7. Bioaccessibility Index

The percentage of bioaccessible phenolic compounds during gastrointestinal digestion was calculated using the bioaccessibility index according to Andrade et al. [43], based on the following formulas:

*C_D_* is the content of each phenolic compound (mg/g) in the soluble fractions during in vitro gastrointestinal digestion, and *C_U_* is the phenolic content (mg/g) in the samples before digestion.

$$Bioaccessibility\;\left(\%\right)=\frac{C_{D}}{C_{U}}\times 100$$

### 3.8. Statistical Analysis

Statistical analyses were performed using IBM SPSS Statistics 19.0. Results were expressed as the mean ± standard deviation. One-way ANOVA was applied, followed by a Duncan’s multiple range test for mean comparisons and difference significance analysis (*p* < 0.05).

## 4. Conclusions

The findings of this study demonstrate that the stability and bioaccessibility of phenolic compounds in purple rice bran extracts (Hom Nil and Riceberry) are significantly affected by in vitro simulated gastrointestinal digestion. Both varieties of rice bran exhibited a notable reduction in antioxidant activity, total phenolic content (TPC), total flavonoid content (TFC), and total anthocyanin content (TAC) across the digestion stages. Specifically, the antioxidant activity measured by DPPH and FRAP assays decreased progressively from the crude extracts to the intestinal phase. Phenolic acids, such as gallic, protocatechuic, vanillic, *ρ*-coumaric, and ferulic acids, showed varying degrees of stability, with sinapic acid remaining notably stable throughout digestion. Flavonoids, including quercetin, rutin, and myricetin, also experienced reductions, with quercetin displaying relative stability in the intestinal phase.

On the other hand, anthocyanins demonstrated significant degradation, particularly during the intestinal phase, underscoring their sensitivity to pH changes. The bioaccessibility index (BI) for most phenolic compounds decreased through the digestive stages, indicating a reduction in the proportion of these compounds available for absorption. This study highlights the importance of considering the stability and bioaccessibility of phenolic compounds in the development of functional foods and nutraceuticals. Future research should explore advanced delivery systems and formulations to enhance the stability and bioavailability of these compounds during digestion, thereby maximizing their potential health benefits. Controlled food delivery and encapsulation technologies may provide viable solutions to improve the efficacy of phenolic extracts from purple rice brans. Overall, the study provides valuable insights into the digestive stability of phenolic compounds in purple rice brans, contributing to the optimization of plant-based bioactives for improved human health.

## Figures and Tables

**Table 1:** Phenolic compounds (mg/g) of purple rice bran extracts during simulated in vitro digestion.

Rice Samples	Phenolic Compounds	Concentration (mg/g)			
		Pre-Digest Phase	Oral Phase	Gastric Phase	Intestinal Phase
Hom Nil	Phenolic acids				
	Gallic acid	0.64 ± 0.02 ^a^	0.56 ± 0.01 ^bc^	0.54 ± 0.03 ^bcd^	0.57 ± 0.03 ^b^
	Protocatechuic acid	1.47 ± 0.05 ^a^	1.18 ± 0.04 ^bc^	1.10 ± 0.07 ^c^	0.81 ± 0.05 ^d^
	Vanillic acid	0.72 ± 0.03 ^ab^	0.63 ± 0.03 ^b^	0.61 ± 0.09 ^b^	0.63 ± 0.08 ^b^
	*ρ*-Coumaric acid	0.29 ± 0.01 ^a^	0.26 ± 0.00 ^b^	0.13 ± 0.02 ^c^	0.13 ± 0.01 ^c^
	Ferulic acid	0.32 ± 0.03 ^a^	0.16 ± 0.02 ^b^	0.18 ± 0.06 ^b^	0.21 ± 0.03 ^b^
	Sinapic acid	0.52 ± 0.04 ^a^	0.51 ± 0.03 ^a^	0.44 ± 0.03 ^b^	0.52 ± 0.03 ^a^
	Total	3.97 ± 0.11 ^a^	3.30 ± 0.09 ^b^	3.01 ± 0.08 ^cd^	2.88 ± 0.07 ^d^
	Flavonoids				
	Rutin	0.55 ± 0.03 ^a^	0.42 ± 0.02 ^b^	0.39 ± 0.04 ^b^	0.25 ± 0.03 ^c^
	Myricetin	2.23 ± 0.06 ^a^	1.63 ± 0.05 ^c^	1.78 ± 0.10 ^b^	1.45 ± 0.08 ^d^
	Quercetin	1.53 ± 0.08 ^a^	1.38 ± 0.07 ^ab^	1.40 ± 0.10 ^ab^	1.45 ± 0.09 ^ab^
	Total	4.31 ± 0.15 ^a^	3.42 ± 0.12 ^bc^	3.58 ± 0.13 ^b^	3.15 ± 0.12 ^c^
	Anthocyanins				
	Cyanidin-3-glucoside	139.41 ± 3.51 ^b^	118.49 ± 3.93 ^c^	156.14 ± 1.99 ^a^	62.73 ± 2.28 ^d^
	Peonidin-3-glucoside	17.96 ± 0.89 ^b^	17.60 ± 1.11 ^b^	22.45 ± 0.52 ^a^	8.62 ± 0.63 ^c^
	Malvidin-3-glucoside	0.35 ± 0.03 ^b^	0.33 ± 0.04 ^b^	0.43 ± 0.02 ^a^	0.23 ± 0.03 ^c^
	Total	157.72 ± 3.70 ^b^	136.43 ± 3.19 ^c^	179.02 ± 4.20 ^a^	71.58 ± 1.68 ^d^
Riceberry	Phenolic acids				
	Gallic acid	0.62 ± 0.01 ^a^	0.53 ± 0.01 ^bc^	0.52 ± 0.03 ^c^	0.54 ± 0.03 ^bc^
	Protocatechuic acid	1.49 ± 0.04 ^a^	1.23 ± 0.03 ^b^	1.15 ± 0.06 ^bc^	0.85 ± 0.04 ^d^
	Vanillic acid	0.77 ± 0.03 ^a^	0.68 ± 0.03 ^ab^	0.67 ± 0.08 ^ab^	0.67 ± 0.10 ^ab^
	*ρ*-Coumaric acid	0.32 ± 0.01 ^a^	0.30 ± 0.00 ^ab^	0.18 ± 0.02 ^c^	0.17 ± 0.01 ^c^
	Ferulic acid	0.40 ± 0.02 ^a^	0.21 ± 0.02 ^cd^	0.22 ± 0.03 ^cd^	0.27 ± 0.04 ^bc^
	Sinapic acid ^NS^	0.53 ± 0.04	0.53 ± 0.04	0.51 ± 0.03	0.54 ± 0.03
	Total	4.14 ± 0.07 ^a^	3.47 ± 0.06 ^b^	3.25 ± 0.08 ^c^	3.04 ± 0.07 ^d^
	Flavonoids				
	Rutin	0.54 ± 0.03 ^a^	0.40 ± 0.01 ^b^	0.38 ± 0.03 ^b^	0.25 ± 0.02 ^c^
	Myricetin	2.03 ± 0.05 ^a^	1.52 ± 0.04 ^c^	1.67 ± 0.07 ^b^	1.26 ± 0.06 ^d^
	Quercetin	1.40 ± 0.07 ^ab^	1.33 ± 0.08 ^b^	1.29 ± 0.09 ^b^	1.35 ± 0.10 ^b^
	Total	3.97 ± 0.22 ^a^	3.25 ± 0.12 ^c^	3.34 ± 0.18 ^bc^	2.86 ± 0.16 ^d^
	Anthocyanins				
	Cyanidin-3-glucoside	50.75 ± 2.98 ^b^	41.62 ± 1.58 ^c^	58.36 ± 1.63 ^a^	24.36 ± 0.95 ^d^
	Peonidin-3-glucoside	8.97 ± 0.87 ^a^	8.88 ± 0.43 ^a^	10.95 ± 0.51 ^b^	4.67 ± 0.27 ^c^
	Malvidin-3-glucoside	0.32 ± 0.03 ^b^	0.31 ± 0.02 ^b^	0.41 ± 0.02 ^a^	0.21 ± 0.01 ^c^
	Total	60.05 ± 2.33 ^b^	50.81 ± 1.97 ^c^	69.72 ± 2.70 ^a^	29.23 ± 1.13 ^d^

Values are expressed as means ± standard deviation. Means with different letters in the same rows were significantly different at *p* < 0.05. ^NS^ indicates non-significant.

**Table 2:** Bioaccessibility index of phenolic compounds in purple rice bran extracts during simulated in vitro digestion.

Rice Samples	Phenolic Compounds	Bioaccessibility Index (%)		
		Oral Phase	Gastric Phase	Intestinal Phase
Hom Nil	Phenolic acids			
	Gallic acid ^NS^	87.06 ± 4.05	84.05 ± 3.48	89.05 ± 3.69
	Protocatechuic acid	80.03 ± 2.68 ^a^	75.07 ± 4.03 ^a^	55.05 ± 2.96 ^b^
	Vanillic acid ^NS^	87.20 ± 7.26	85.15 ± 6.02	88.15 ± 6.23
	*ρ*-Coumaric acid	90.03 ± 3.05 ^a^	45.05 ± 2.64 ^b^	45.05 ± 2.64 ^b^
	Ferulic acid ^NS^	50.63 ± 9.78	55.69 ± 10.76	65.82 ± 12.72
	Sinapic acid ^NS^	98.78 ± 15.18	85.49 ± 10.95	100.93 ± 13.24
	Total	83.13 ± 3.01 ^a^	75.84 ± 3.83 ^b^	72.58 ± 3.73 ^b^
	Flavonoids			
	Rutin	75.03 ± 2.63 ^a^	70.21 ± 6.62 ^a^	45.14 ± 4.25 ^b^
	Myricetin	73.07 ± 3.93 ^a^	80.06 ± 3.78 ^a^	65.05 ± 3.07 ^b^
	Quercetin ^NS^	90.33 ± 9.50	92.27 ± 8.71	95.27 ± 8.99
	Total ^NS^	79.41 ± 5.61	83.07 ± 5.12	73.15 ± 4.56
	Anthocyanins			
	Cyanidin-3-glucoside	85.07 ± 4.24 ^b^	112.07 ± 4.84 ^a^	45.03 ± 1.94 ^c^
	Peonidin-3-glucoside	98.01 ± 1.96 ^b^	125.32 ± 11.01 ^a^	48.12 ± 4.23 ^c^
	Malvidin-3-glucoside	95.94 ± 16.43 ^a^	122.88 ± 17.45 ^a^	65.47 ± 9.30 ^b^
	Total	86.57 ± 4.02 ^b^	113.56 ± 4.51 ^a^	45.41 ± 1.81 ^c^
Riceberry	Phenolic acids			
	Gallic acid ^NS^	84.46 ± 2.27	83.22 ± 7.42	86.23 ± 7.69
	Protocatechuic acid	82.17 ± 6.33 ^a^	77.16 ± 5.94 ^a^	57.12 ± 4.40 ^b^
	Vanillic acid ^NS^	89.24 ± 18.71	89.24 ± 18.71	89.24 ± 18.71
	*ρ*-Coumaric acid	93.39 ± 10.34 ^a^	56.24 ± 6.22 ^b^	53.22 ± 5.89 ^b^
	Ferulic acid ^NS^	55.03 ± 12.40	56.05 ± 12.63	69.29 ± 15.61
	Sinapic acid ^NS^	99.33 ± 9.70	96.32 ± 9.41	101.33 ± 9.90
	Total	83.87 ± 2.25 ^a^	78.66 ± 1.98 ^b^	73.55 ± 2.30 ^c^
	Flavonoids			
	Rutin	74.36 ± 9.04 ^a^	71.34 ± 8.67 ^a^	47.23 ± 5.74 ^b^
	Myricetin	75.19 ± 6.58 ^a^	82.21 ± 7.19 ^a^	62.16 ± 5.44 ^b^
	Quercetin ^NS^	95.05 ± 6.43	92.46 ± 11.24	96.48 ± 11.73
	Total ^NS^	82.06 ± 6.75	84.30 ± 8.20	72.21 ± 7.22
	Anthocyanins			
	Cyanidin-3-glucoside	82.13 ± 5.52 ^b^	115.18 ± 7.98 ^a^	48.08 ± 3.33 ^c^
	Peonidin-3-glucoside	99.33 ± 10.14 ^b^	122.39 ± 11.81 ^a^	52.17 ± 5.03 ^c^
	Malvidin-3-glucoside	97.48 ± 11.81 ^b^	126.62 ± 15.34 ^a^	64.31 ± 7.79 ^c^
	Total	84.75 ± 5.76 ^b^	116.28 ± 7.91 ^a^	48.76 ± 3.31 ^c^

Values are expressed as means ± standard deviation. Means with different letters in the same rows were significantly different at *p* < 0.05. ^NS^ indicates non-significant.

## Data Availability

The data presented in this study are available on request from the corresponding author.
